# Data Validation and Verification Using Blockchain in a Clinical Trial for Breast Cancer: Regulatory Sandbox

**DOI:** 10.2196/18938

**Published:** 2020-06-02

**Authors:** Tomonobu Hirano, Tomomitsu Motohashi, Kosuke Okumura, Kentaro Takajo, Taiyo Kuroki, Daisuke Ichikawa, Yutaka Matsuoka, Eisuke Ochi, Taro Ueno

**Affiliations:** 1 SUSMED Inc Tokyo Japan; 2 Division of Health Care Research Center for Public Health Sciences National Cancer Center Japan Tokyo Japan; 3 Faculty of Bioscience and Applied Chemistry Hosei University Tokyo Japan

**Keywords:** blockchain, clinical trial, data management, validation, breast cancer, regulatory sandbox

## Abstract

**Background:**

The integrity of data in a clinical trial is essential, but the current data management process is too complex and highly labor-intensive. As a result, clinical trials are prone to consuming a lot of budget and time, and there is a risk for human-induced error and data falsification. Blockchain technology has the potential to address some of these challenges.

**Objective:**

The aim of the study was to validate a system that enables the security of medical data in a clinical trial using blockchain technology.

**Methods:**

We have developed a blockchain-based data management system for clinical trials and tested the system through a clinical trial for breast cancer. The project was conducted to demonstrate clinical data management using blockchain technology under the regulatory sandbox enabled by the Japanese Cabinet Office.

**Results:**

We verified and validated the data in the clinical trial using the validation protocol and tested its resilience to data tampering. The robustness of the system was also proven by survival with zero downtime for clinical data registration during a Amazon Web Services disruption event in the Tokyo region on August 23, 2019.

**Conclusions:**

We show that our system can improve clinical trial data management, enhance trust in the clinical research process, and ease regulator burden. The system will contribute to the sustainability of health care services through the optimization of cost for clinical trials.

## Introduction

Clinical trials involve a large flow of medical information, and it is necessary to secure the transparency and traceability of clinical data. The International Council for Harmonisation of Technical Requirements for Pharmaceuticals for Human Use–Good Clinical Practice (ICH-GCP) provides guidance for monitoring the conduct of a clinical trial to verify that reported clinical data are complete, accurate, and accounted for by source records. The long-standing practice in the pharmaceutical and medical device industries has been frequent site visits and 100% source data verification (SDV) on site to ensure that data captured in source records are transferred correctly to the case report forms. It has been estimated that clinical trial monitoring by SDV can consume one-quarter of the sponsor’s entire budget for a clinical trial [1]. As the complexity and size of clinical trials grow, it has become increasingly expensive to apply the 100% SDV approach [2,3]. For regulators of trials, such as the US Food and Drug Administration (FDA), European Medicines Agency (EMA) in the European Union, and Pharmaceuticals and Medical Devices Agency (PMDA) in Japan, data auditing is challenging since there is no easy and secure way to access or view complex data. As such, process improvement is an active area of research [4]. With the involvement of more parties and more exchanges, the risk for human-induced error, whether unintentional or malicious, will increase. Previous studies have shown that 17% of authors of clinical trials reported they were personally aware of intentional fabrication in research [5,6]. In the case of misconduct in a clinical trial by an employee of Novartis Pharma, clinical data were manipulated to attribute qualities to the hypertension drug valsartan that it did not possess, such as preventing stroke [7]. The fabricated clinical data were used in an advertising campaign for the drug, and patients were prescribed the drug based on incorrect information. Following misconduct in clinical trials, the Japanese government established and enforced the Clinical Trials Act in 2018 [8]. It obligates clinical researchers to monitor and assure quality, observe practice criteria, and manage conflicts of interest.

Blockchain is emerging as a groundbreaking technology for secure data control in different areas. Bitcoin was the first application of blockchain as a digital currency in extensive use [9]. Recently, researchers have started to focus on using blockchain methodology for building a cryptographic proof of medical systems [10]. They have applied blockchain technology in various health care systems, with potential applications in supply chain management of health care products [11-13], insurance claims processing [14], management of electronic health records and electronic medical records [15-18], maintenance of protocols in clinical trials [19-21], and data management in clinical trials [22-25]. Although there can be many applications of blockchain technology, it is necessary that blockchain be an appropriate technical solution to a particular problem. If there are no incentives for data tampering and all writers can be trusted, blockchain technology is not necessary. On the other hand, if there are incentives for data tampering and it costs a lot to use a trusted third party, such as the Clinical Research Organization, using blockchain makes sense [26]. Due to the frequent occurrences of misconduct in clinical trials and the large consumptions of clinical trial budgets and time by current SDV practices, blockchain is an appropriate technical application in the data management of clinical trials to solve these problems.

We propose a solution to challenges in the current clinical trial system by using blockchain technology coupled with technologies such as client hashchain, encryption protocol, and health check function in servers for hazard management. The system was used in a clinical trial at the National Cancer Center of Japan that investigated the effect of home-based high-intensity interval training for breast cancer survivors. The project was conducted to demonstrate clinical data management using blockchain technology under the regulatory sandbox of the Japanese Cabinet Office [27,28]. This sandbox allows organizations to apply for demonstration and evaluation of new technology, such as blockchain and Internet of Things, without being subject to existing regulations. It simultaneously opens up the possibility for future deregulation measures. During the trial, we experienced the disruption of Amazon Web Services (AWS) cloud servers in the Tokyo region on August 23, 2019 [29]. We report the effect of the blockchain network and health checkup function, which resulted in system survival with zero downtime and secure clinical data registration during the hazardous server shutdown event.

## Methods

### Breast Cancer Clinical Trial

A clinical trial that investigates the effect of intervention by home-based high-intensity interval training for breast cancer patients was conducted. The study was a parallel-group, single-blind randomized controlled trial. Patients were randomly assigned to the active group with the habit-B program (high-intensity interval training, exercise counseling and guidance, home-based exercise support using information and communication technology, and a wearable device) or treatment as usual with a wearable device.

The eligibility criteria for participants were (1) female, aged between 20 and 59 years at diagnosis; (2) diagnosed with stages I to IIa breast cancer and currently 2 to 13 months postsurgical treatment; (3) not requiring cancer chemotherapy aside from hormone therapy; (4) ability to read, write, and understand Japanese; (5) ability to complete an electronic Patient Reported Outcome Questionnaire via smartphone; (6) consent to trial participation obtained in writing from the patient themselves; and (7) currently engaging in not more than moderate intensity exercise for 30 minutes on two separate days per week (total of 60 minutes).

The researcher provided computer-generated random allocation. Using the generated account, the contents of the app that participants used during the clinical trial were assigned automatically to either the habit-B program or the control. When a participant first visits the hospital, she is randomly assigned to an intervention, either with the habit-B program or treatment as usual with a wearable device, after signing informed consent. An independent data center provides computer-generated random allocation as a log-in account for the app. Based on the allocation sequences, the contents of the app participants use during the trial are assigned automatically to either the habit-B program or the control.

The study received ethical approval from the institutional review board of the National Cancer Center Japan and was registered at University Hospital Medical Information Network–Clinical Trial Registry [UMIN000036400]. Participants provided written informed consent to take part in the study. The study began in May 2019 and is ongoing [30].

### Architecture of the Data Management System

**Figure 1:**
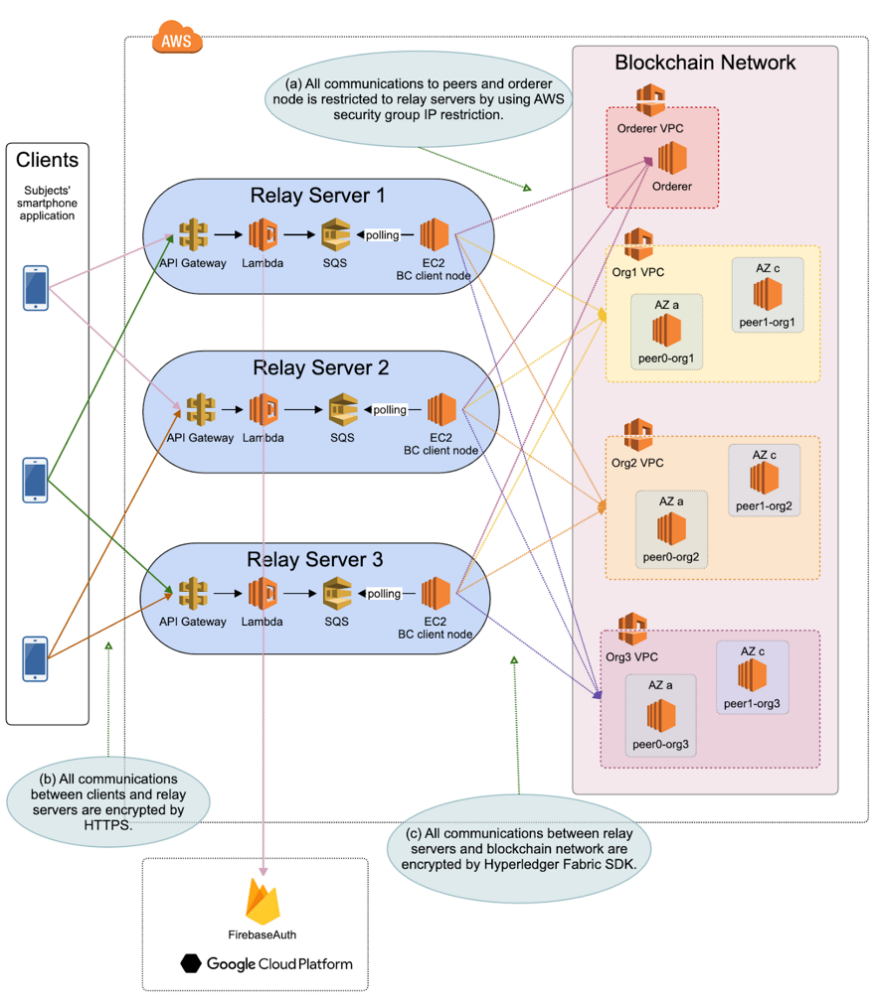
Data management system including client smartphones, relay servers, and blockchain network. The collected data from patient smartphones were sent to the blockchain network via relay servers. We used three relay servers, and the app randomly selected two relay servers to send the data after the authentication of the client device. We have configured the blockchain network to span multiple availability zones in Amazon Web Services.

The data management system that collects the clinical trial data consists of client smartphones, relay servers, and a blockchain network (Figure 1). The relay servers and blockchain network are built on AWS in the Tokyo region. We use Hyperledger Fabric v1.0 (Linux Foundation), an open-source blockchain platform that has been widely used, to operate the blockchain network. Hyperledger Fabric enables throughput of more than 3500 transactions per second in popular deployment configurations [31].

Data collected from patient smartphones are sent to the blockchain network via relay servers. We use three relay servers, and the app randomly selects two relay servers to send the data after the authentication of the client device. The client app contains an authentication key, and the relay servers verify the key so that the relay server only accepts access from the app for the clinical trial. The relay servers also verify the account for the app using Firebase authentication. These communications between clients and relay servers are encrypted using https. By deploying the relay server and setting the blockchain software development kits to append-only mode, the relay servers send the received data to the blockchain network. The data sent from the relay servers to the blockchain network are encrypted using the authentication protocol by the Hyperledger Fabric. By configuring the internet protocol address restriction to the listed relay proxy, the blockchain network, which contains the clinical trial data, is protected against malicious attack from the external network. The blockchain network is made up of three organizations, each of which contains two validating peers. Each identification and password for relay servers, blockchain network organizations, and nodes of the blockchain network is managed by two independent departments in SUSMED Inc and the National Cancer Center of Japan [22].

We have configured the blockchain network to span multiple availability zones (AZs) in AWS. Each AZ is made up of one or more data centers equipped with independent power, cooling, and networking to ensure fault tolerance. We have also implemented a periodic health check function using the Amazon EC2 Auto Scaling Service. The function continues to maintain a fixed number of instances even if an instance becomes unhealthy. If an instance becomes unhealthy, the group terminates the unhealthy instance and launches another instance to replace it.

In order to increase system durability and prevent data loss, we have configured two kinds of queues in our system. The mobile app puts clinical data into the queue before sending, dequeues and sends the message, and then removes the data from the queue. If relay servers are unavailable, the sending process fails and the data remain available in the queue until the relay servers process it. The other queue is in a relay server system, and the queue is configured using AWS Simple Queue Service (SQS). When a relay server system receives the clinical data, the data are enqueued. In the relay server system, an EC2 instance is responsible for dequeuing and sending the message to the blockchain network. The data are removed from the queue after being successfully processed by the blockchain network. Otherwise, the data remain available in the queue until the sending process succeeds or the SQS retention period expires. In this system, we set the SQS retention period to 7 days. In doing so, we have seven days’ time to recover the blockchain network if there is a breakdown.

### Data Collection Using Smartphone

The Global Physical Activity Questionnaire is an internationally standardized questionnaire for surveying physical activity level [32-34]. Fear of cancer recurrence is assessed by the overall fear index score on the Concerns About Recurrence scale [35,36]. Depression is assessed using the Patient Health Questionnaire–9 [37]. Fatigue is assessed by the Cancer Fatigue Scale. Sleep is assessed by the Athens Insomnia Scale [38]. Quality of life is assessed using the EuroQol 5-Dimensions questionnaire [39,40]. All data listed above are stored in JavaScript Object Notation format in the database.

### Data Registration to the Blockchain Network

When a participant first logs in to the system, the app generates a secret hash key and preserves it in the client device until the end of the study. The client device calculates a hash value based on the data generated by the patient and the secret hash key as well as the previous hash value using the SHA-256 hash algorithm [22,41]. Thus, the hash value in the client device comprises the chain structure. The hash value is also registered in the blockchain network along with the clinical data in order to guarantee tamper resistance of the value, although the secret hash key was preserved in the client device until the end of the study. The collected data and client hash value are sent to the blockchain network via relay servers. We use three relay servers, and the app randomly selects two relay servers to send the data after the authentication of the client device. Client nodes of the blockchain network are settled in the relay servers and the data are sent from the relay server to the blockchain network. The transactions are validated and accepted by the blockchain network in the following processes:

- Proposal: transaction is sent from client app to the endorsers in each organization
- Endorse: each endorser verifies that (1) the transaction proposal is well formed, (2) it has not already been submitted, (3) the signature is valid, and (4) the client is properly authorized to perform the proposed operation, which is described in the chaincode. If the transaction is validated, the chaincode is executed and the result with the signature is returned to the client
- Submit: client verifies that the number of signatures from the organizations satisfies the endorsement policy. If it is satisfied, the transaction is sent to the ordering service, which orders the series of transactions in chronological order and creates the block of transactions
- Broadcast: the block is delivered to all nodes
- Commit: if each block is well formed and validated to fulfill the endorsement policy, the block is appended to the chain in each node [22]

### Data Validation and Verification

When participants complete the data input for the clinical trial, it is necessary to verify all data records sent to the blockchain network to satisfy the following conditions:

- Blockchain network has received all required data types for each participant
- All data records for each participant are correct as a client hashchain
- Blockchain network has received clinical records from two relay servers for each record, and the records are the same

The first condition means that the blockchain network has received all records that are expected to be sent from clients. There are three types of records in the clinical trial. To begin with, a client sends a record that indicates that a secret hash key has been generated. When clinical data are created, the data are sent to the blockchain network via two relay servers. Finally, when the participants complete the clinical trial, the client sends the secret hash key to the blockchain network.

The second condition verifies that all data records registered to the blockchain network are correctly linked as a client hashchain. We sort all data records by their generated time and make a hash value from the clinical data, the secret hash key preserved in the client device until the end of the study, and the previous hash value. Then, we compare the results with the hash value that has been registered to the blockchain network. Due to the sensitivity of a hash function output in relation to its input, changing the data will result in a completely different hash string. Therefore, we can confirm these hash values are generated by the client device that preserves the secret hash key. By verifying this condition, we can detect if a malicious attacker has falsified the clinical data using hash values and clinical data that were stolen.

The purpose of the last condition is to confirm that the relay servers have not been hacked. Clients send their clinical data to two relay servers that are randomly selected from the three relay servers. If malicious users hack a relay server and they send some data from the relay server, the blockchain network will store the data. In order to detect the fraud access and verify the data, we need to confirm that all clinical data is received from two relay servers simultaneously and the data records are identical.

### Data Availability

The data that support the findings of this study are available from the corresponding author upon reasonable request.

## Results

### Registration and Assignment

**Figure 2:**
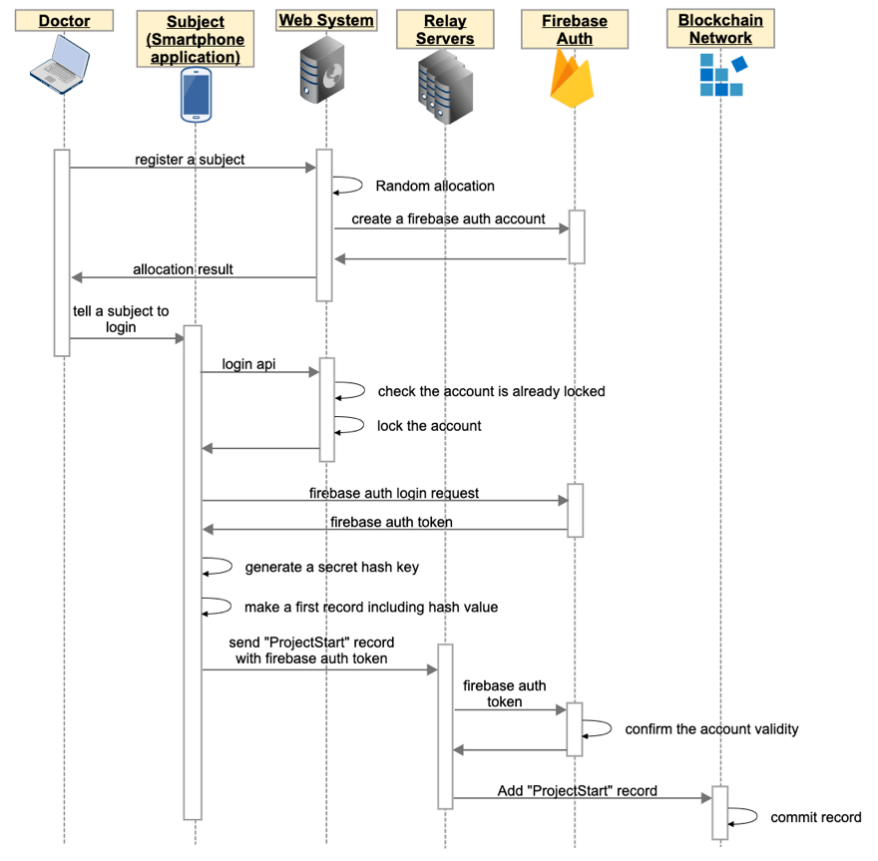
When a participant first visits the hospital, a medical doctor decides if they fit the eligibility criteria. After obtaining informed consent, the doctor registers the patient to the clinical trial using a web-based allocation system. The system automatically creates an account for the participant, and the doctor gives the account to the participant. The participant downloads the app to their smartphone, and they can log in to the app using the account created by the doctor. When the participant first logs in to the app, the account is locked to prevent impersonation, and the secret hash key is then generated. The secret hash key will be preserved in the client app until the participant completes the trial. Using the secret hash key in the client device, the app calculates a hash value. The app sends the first record to the blockchain network via relay servers, and the data is registered in the tamper-resistant blockchain system.

The participant downloads the app to their own smartphone through the internet and logs in to the system using the account provided by the researcher. When the participant first logs in to the system, the app generates a secret hash key and preserves it in the client’s device until the end of the study. The account is locked when the participant first logs in to the system in order to prevent impersonation by stolen account or brute force attack (Figure 2).

### Data Collection

**Figure 3:**
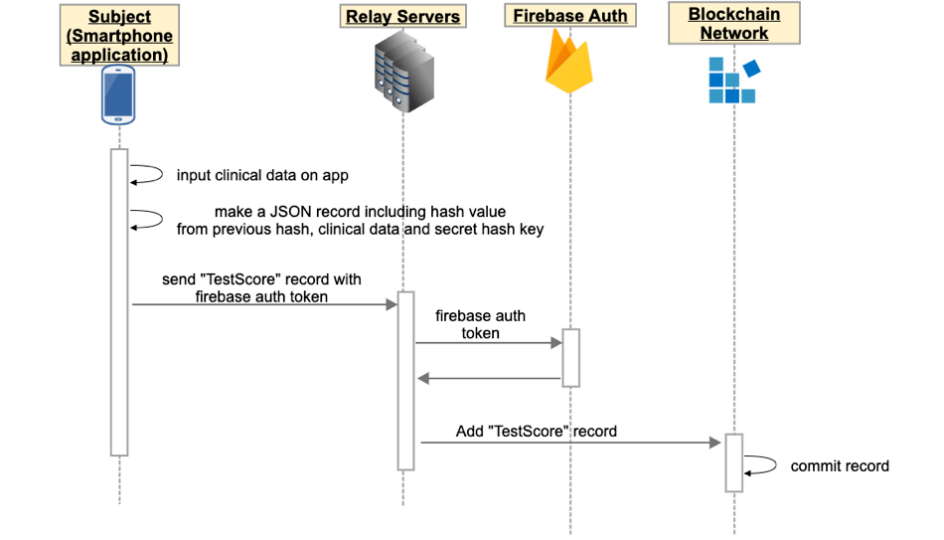
During the clinical trial, participant inputs clinical data using the mobile app. The app calculates the hash value based on the clinical data, the secret hash key preserved in the smartphone until the end of the study, and the previous hash value. The app sends the clinical data and a hash value to the blockchain network via relay servers.

During the trial, the participant inputs the data using the app, and the data are registered to the blockchain network via relay servers (Figure 3). The secret hash key is preserved only in the client’s device during the clinical trial. The client app calculates a hash value based on the medical data, the secret hash key, and the previous hash value using the SHA-256 hash algorithm. The client hash value is also sent to the blockchain network along with the clinical data.

### Completion of the Clinical Trial

**Figure 4:**
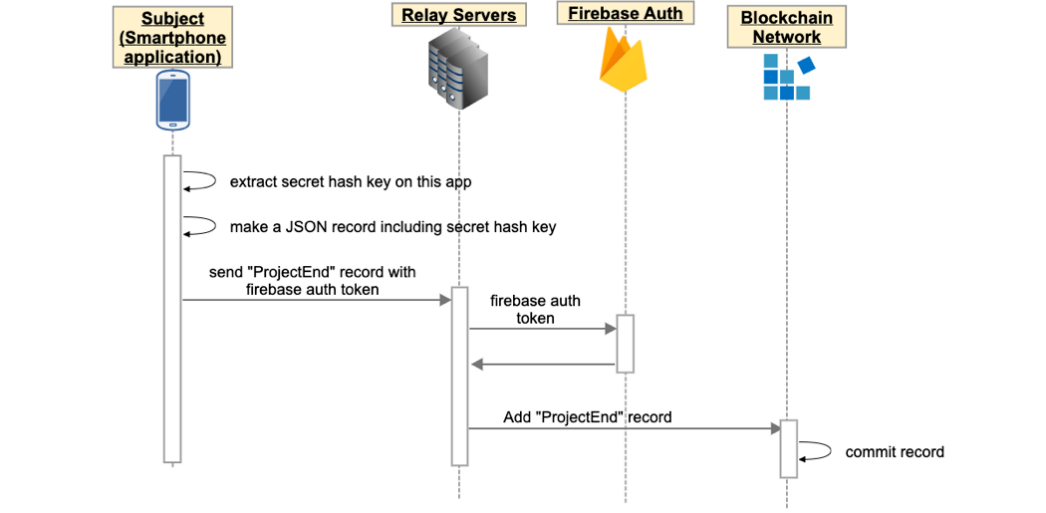
At the end of the clinical trial, the app extracts the secret hash key from the smartphone and sends it to the blockchain network via relay servers.

When the participant completes the clinical trial, the app sends the secret hash key to the blockchain network, and all data of the participant is registered to the blockchain network (Figure 4).

### Data Validation and Verification

We verify and validate the patient’s data collected in the clinical trial using a predetermined protocol. For regulators such as FDA, EMA and PMDA, the full transaction history since the beginning of the study is readily available with precise log data, and the auditing process can be done quickly and with the reliance that all clinical data are guaranteed or version controlled [23].

**Figure 5:**
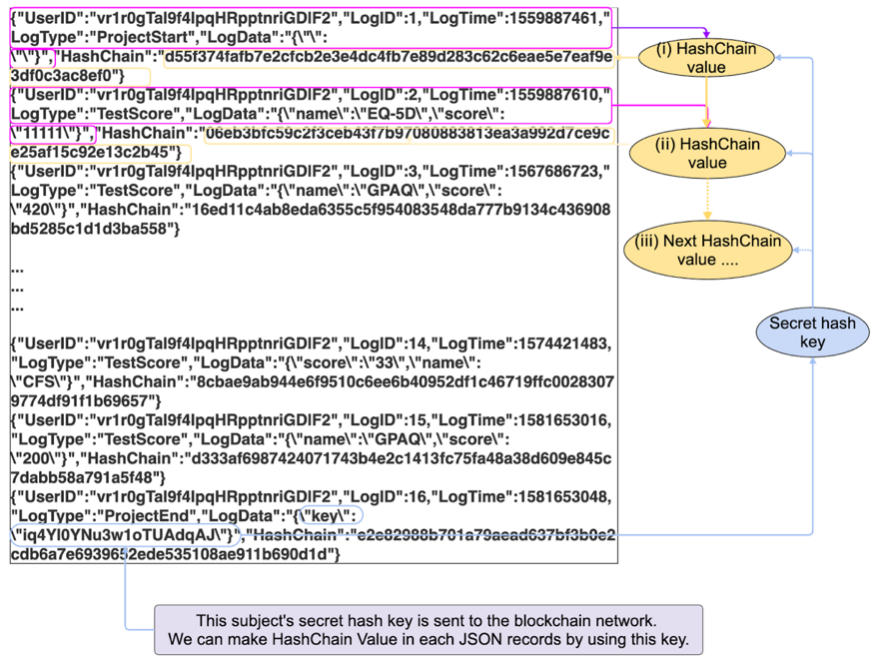
Data verification and validation using client hashchain. The first hash value is made from the first data and secret hash key. Subsequent hash values are made from clinical data, the previous hash key, and secret hash key.

**Figure 6:**
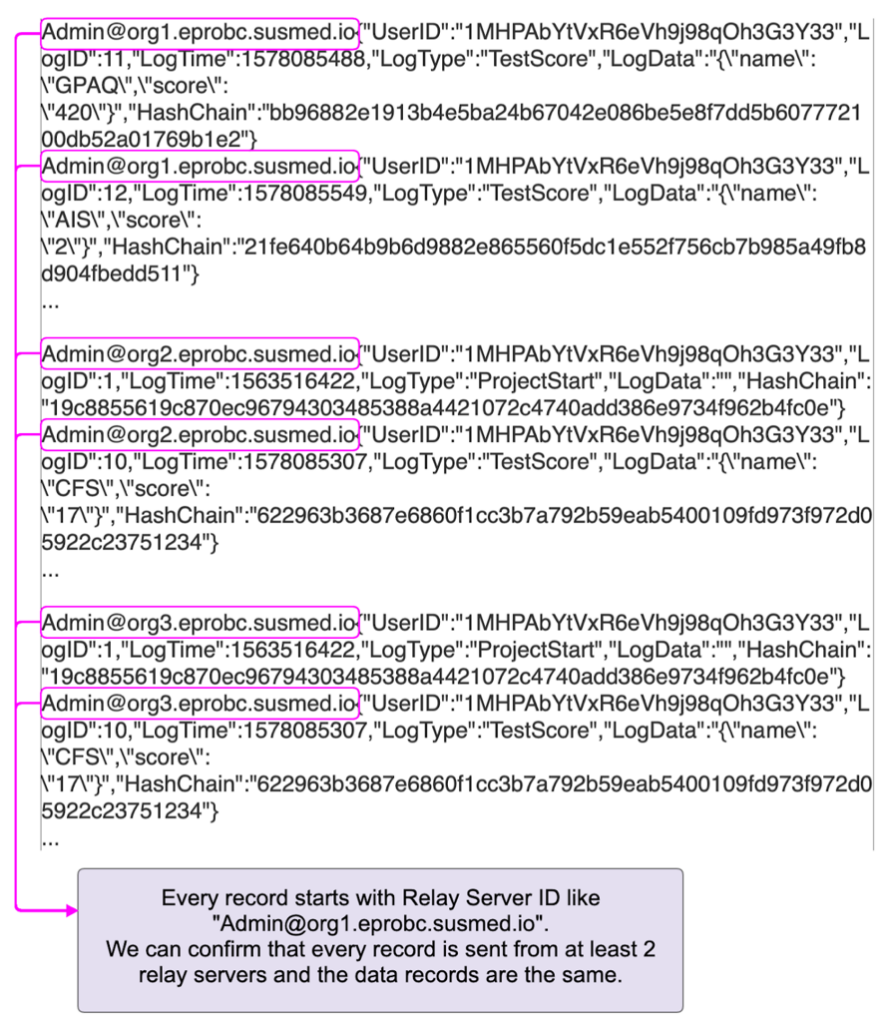
Data verification and validation using records from relay servers.

Blockchain technology ensures a secure and tamper-proof transaction history so that the blockchain network provides an integrity-protected data storage and process transparency [24]. However, there can be vulnerability before registration to the database in the blockchain network. The data can be tampered with and impersonation could occur, resulting in impairment of reliability of the data. To prevent impersonation of the client, we have configured the mechanism so that the account is locked when the participant first logs in to the system. The client app contains the authentication key, and the relay server checks the key so that the relay server only accepts access from the app for the clinical trial. By configuring the internet protocol address restriction to the listed relay server, the blockchain network, which contains the medical data, is protected against external attack. This configuration makes our system highly secure. Even if an attacker breaks the above security system under impersonation or unauthorized access to the relay server, we can validate and distinguish the correct data using the client hashchain function. In order to complement the tamper resistance of the blockchain network and ensure reliability of the entire system, we verified and validated the data integrity using client hashchains and multiple relay servers. User data along with the client hashchain are registered to the blockchain network. The app sends the user data and client hashchain to the blockchain network during the clinical trial. At the end of the trial, the app sends the secret hash key preserved in the client device to the blockchain network. Using the secret hash key, we verify the integrity of the data by calculating the hashchain retrospectively (Figure 5). User data and the client hashchain are sent from multiple relay servers to the blockchain network. A client app sends its clinical data to two relay servers, which are randomly selected from the three relay servers. In order to detect fraudulent access and verify the data sent from the relay servers, the data are compared with each other to confirm that the records are the same (Figure 6).

**Figure 7:**
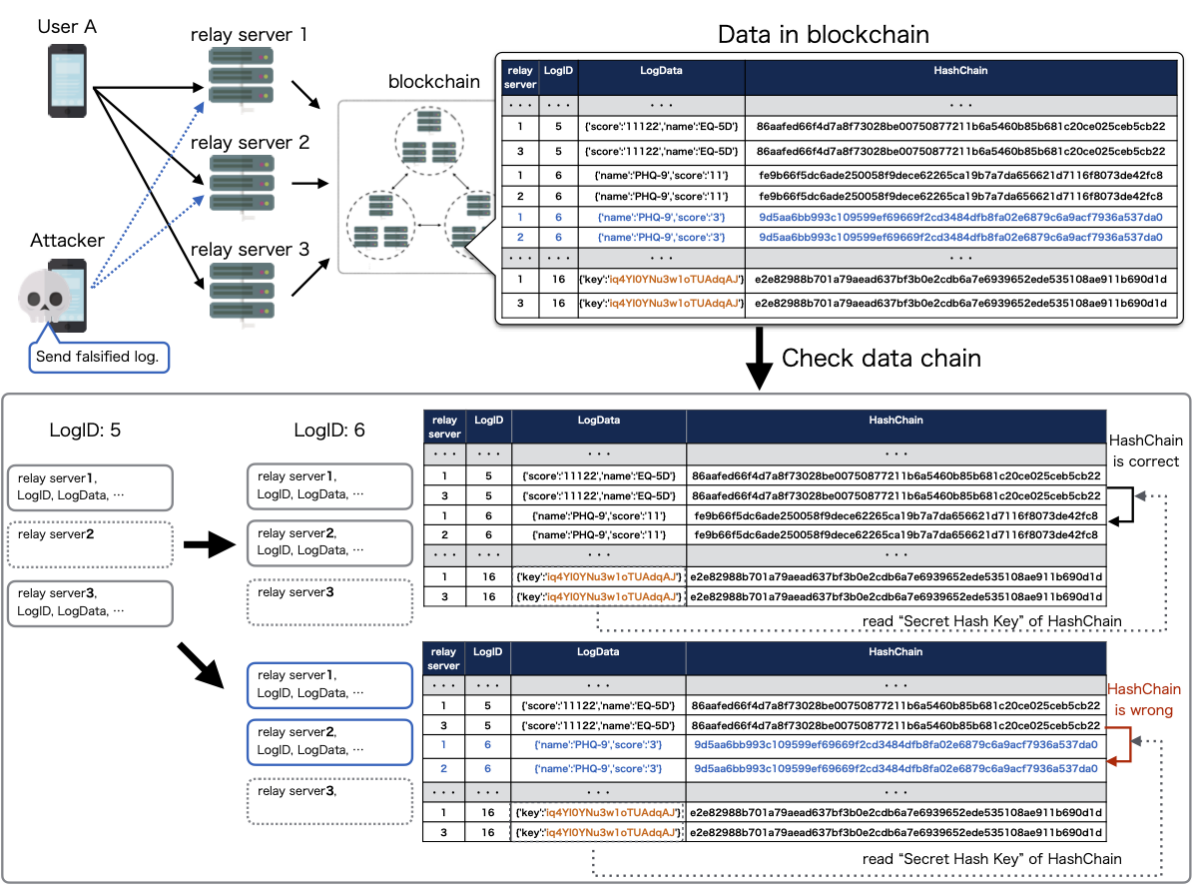
Simulation of data falsification by client impersonation. If an attacker successfully logs into the app using a stolen account or brute force attack, the attacker can create clinical data from another client device. The data from the impersonated client will be denied since only the true participant’s app preserves the secret hash key that is required to calculate the hashchain.

**Figure 8:**
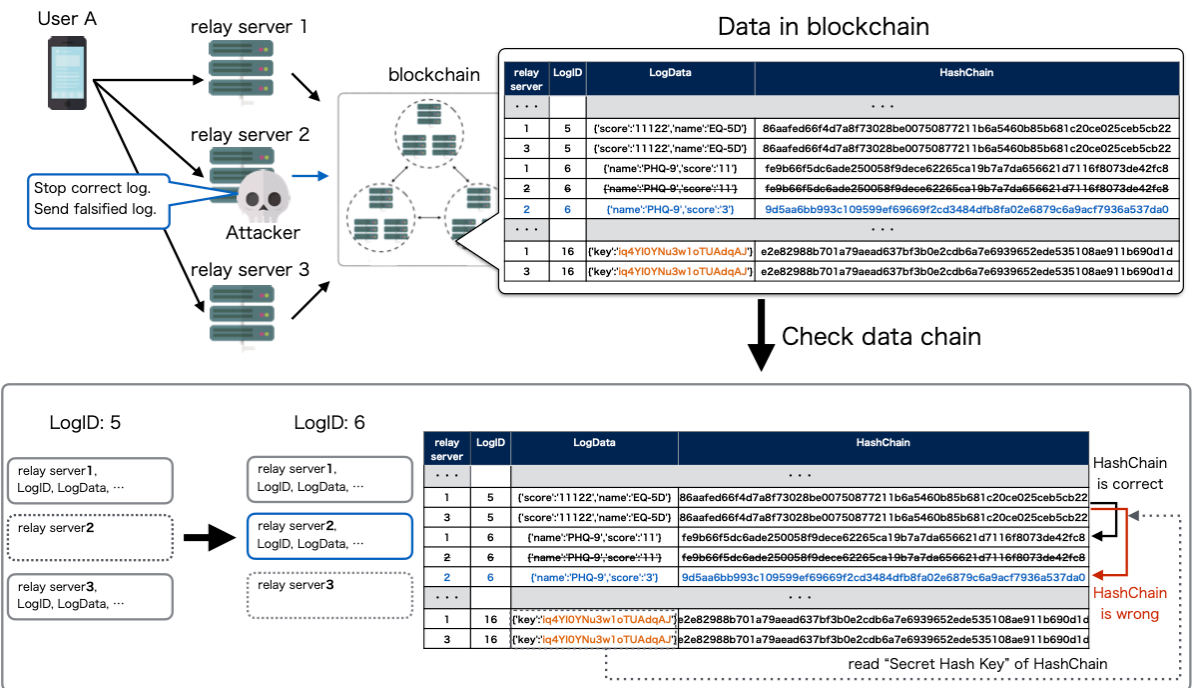
Simulation of data falsification by fraudulent access to the relay server. If an attacker successfully accesses the relay server fraudulently, the attacker can send falsified data to the blockchain. The falsified data from the hacked server will be detected by the integrity of the hashchain.

To evaluate the validation, we have simulated and tested client impersonation and data falsification by fraudulent access to the relay server. The integrity of the data can be maintained even if attacker tries to manipulate the data by impersonating the client device (Figure 7) or fraudulently accessing the relay servers (Figure 8). The result suggests that our proposed function of client hashchain and multiple relay servers can be a complement to the tamper resistance of the blockchain network to ensure the reliability of the entire system.

### Data Integrity During Amazon Web Services Disruption Event

**Figure 9:**
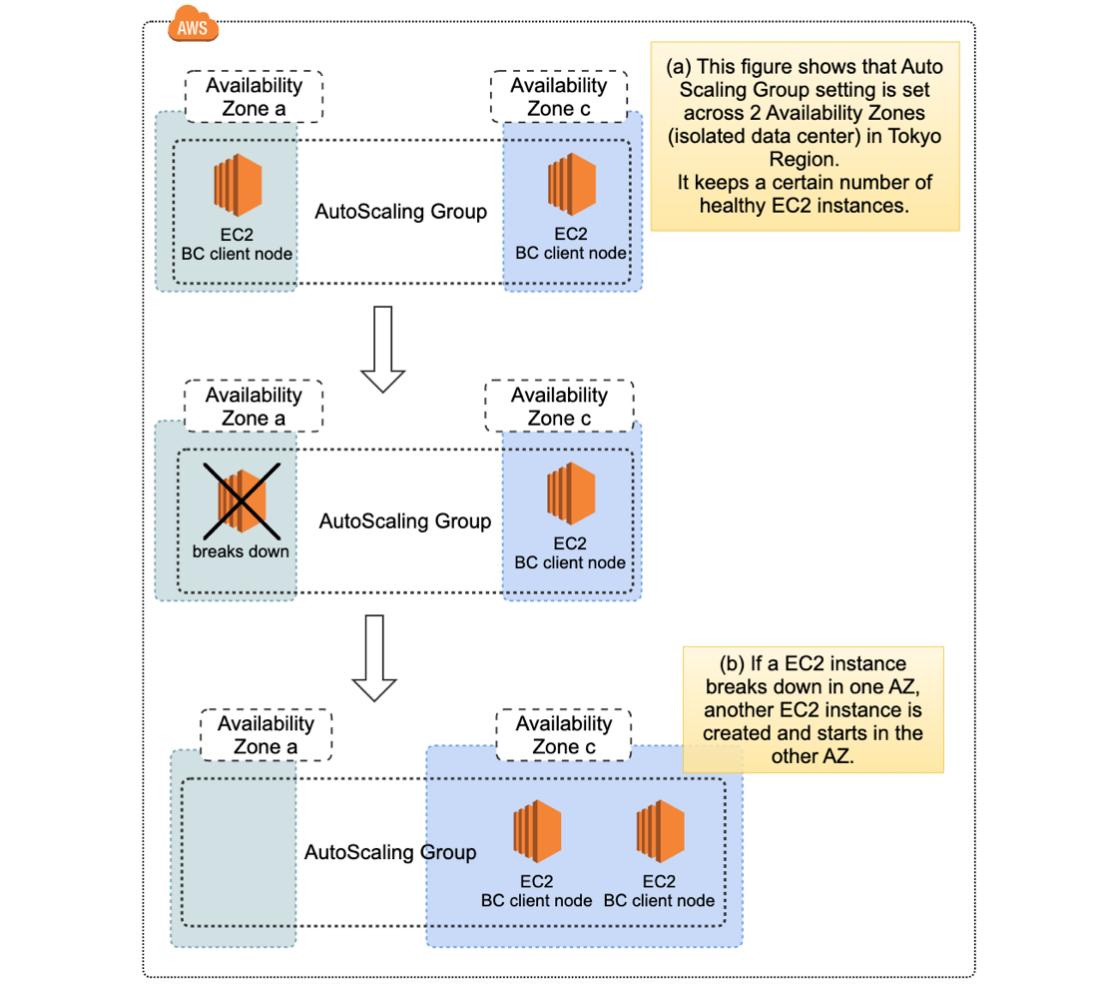
System resilience during Amazon Web Services disruption event in the Tokyo region. To secure the redundancy of the system, we have configured the blockchain network to span multiple availability zones (AZs) in Amazon Web Services. Each AZ is made up of one or more data centers equipped with independent power, cooling, and networking to ensure fault tolerance. We have also implemented a periodic health check function using the Amazon EC2 Auto Scaling Service. When one of the nodes in the blockchain network broke down due to the severe disruption of the data center on August 23, 2019, our system maintained stable operation and lost no data by the redundancy of the blockchain network. Furthermore, the health check system using the Amazon EC2 Auto Scaling Group enabled the automatic recovery of the blockchain node in a healthy AZ after the data center disruption event.

During the trial, we encountered a disruption event of the AWS cloud server in the Tokyo region on August 23, 2019 [29]. This resulted in impaired EC2 instances for some resources in the affected area of the AZ. One of the blockchain network nodes shut down due to disruption of the data center. Since the blockchain network was composed of three organizations that contain two validating peers and we had configured the blockchain network to span multiple AZs in AWS, the redundancy maintained stable operation of the system during the event. Furthermore, our configuration of a periodic health check function using Amazon EC2 Auto Scaling Service automatically restored the affected node in a healthy data center (Figure 9). We have confirmed there were no error records during the disruption event.

Even if further instances in the blockchain network or relay server components break down, we are able to prevent data loss. There are queues in the client smartphone app and relay servers, and the queues retain the clinical data until data are successfully sent. While the queues contain the data, we can recover the affected system.

## Discussion

### Principal Findings

In this study, we showed that a data management system using blockchain technology can reliably protect medical data and provide an immutable and fully traceable audit trail in a clinical trial. Due to the sensitivity of a hash function’s output in relation to its input, any change in clinical data will result in a completely different hash string. This string will be an input of the next block’s hash function, and the resulting string will be completely different from what it was prior to the data modification. As such, data integrity in a blockchain-based data management system can be checked by simply comparing the hash values of a blockchain. Verifying integrity can be done quickly, as the regulator needs only check for hash string equivalence. Our system can thus offer an improvement in clinical trial data management, enhancing trust in the clinical research process and easing regulator burden [23]. Although we used Hyperledger Fabric for the blockchain platform, other open-source platforms such as Ethereum can also be applied [42,43]. Furthermore, our system can also be used in clinical trials that collect data via other devices like PC or Internet of Things.

ICH-GCP provides guidance for monitoring conduct of a clinical trial to verify that reported clinical data are complete, accurate, and accounted for by source records. Representatives from the pharmaceutical and medical device industries must visit hospitals frequently and verify data by SDV to confirm that collected medical data are transferred correctly to the case report forms. It has been reported that almost half of the total budget for a phase 3 clinical trial is GCP-related and half of the GCP-related cost is related to SDV [1,3]. The total cost for current SDV in a typical phase 3 trial is estimated at US $90 million, which will be added to the price of the drug [3]. As the size and complexity of clinical trials grow, the cost of monitoring by the traditional method has become increasingly expensive. EMA and FDA have published updated guidelines for the monitoring and oversight of clinical trials. Both agencies encourage study sponsors to implement remote and risk-based monitoring [44,45]. Although remote monitoring may be an attractive alternative to traditional SDV, some studies have shown that remote monitoring can be more time-consuming than traditional monitoring and may be insufficient as a complete alternative for SDV [46]. Another option, risk-based monitoring, also needs risk assessment before its implementation and will not eliminate the need for data verification by the human eye. Conversely, our blockchain-based system enables secure data management without labor-intensive monitoring operations in medical practice. It can drastically reduce the cost of human resources and the risk of human-induced error. Information technology systems for the management of clinical trials need to keep audit trails permanent, tamper-proof, and verifiable, as stipulated by FDA 21 CFR part 11. Blockchain for clinical research involves a promising set of technologies that may advance data integrity and efficiencies in clinical research.

This study was conducted to demonstrate clinical data management using blockchain technology under certification of the regulatory sandbox of the Japanese Cabinet Office. The Japanese government aims to actively explore innovative and disruptive technologies through this regulatory sandbox system under the Cabinet Secretariat to address challenges such as extreme aging of the population [28]. Japanese society is facing challenges like increased national public health expenditures, higher demand for health care services lacking appropriate cost controls, and shortage of health care workers. Some of these challenges are becoming more acute, with recent data indicating that expenditures in health care insurance are growing in Japan [47]. The data collected through the demonstrations in the regulatory sandbox will be used in deliberation for regulatory reform, which will facilitate the creation of innovative business activities with new technologies and new business models.

Severe disruption of cloud server services occurred in the AWS Tokyo region on August 23, 2019, during the clinical trial. Thousands of companies, ranging from internet retailers and smartphone payment platforms to game providers, were affected and their services were suspended. During the event, customers of these services could not access their own information and lost data that should have been generated during the event. Our system maintained stable operation and lost no data from the participants of the clinical trial during the disruption event due to the redundancy of the blockchain network. The configuration of the blockchain network to span multiple data centers in AWS, along with the health check system using Amazon EC2 Auto Scaling, enabled automatic recovery of the blockchain network after severe disruption of the data center. These features in our system, which provide zero downtime for the availability of patient data, are suitable to meet the requirements of clinical trials.

### Limitations

Further studies are needed to verify the scalability of the system for conducting various kinds of clinical trials. Although the transaction throughput in the Hyperledger Fabric platform that we used is much higher than public blockchain, further studies like multiple clinical trials in a single system may be fruitful. Data collection via multiple devices should also be tested toward the dissemination of virtual clinical trials.

### Conclusion

In our study, we ran a clinical trial for breast cancer using a blockchain-based clinical data management system. The data were validated by protocol, and traceability was secured. Even with the severe disruption event of cloud server services, our system preserved the integrity of the clinical trial data. The study was conducted as a regulatory sandbox project by the Cabinet Office of Japan and will open the possibility for future deregulation measures enabling efficient clinical development.

## Abbreviations

AWS: Amazon Web Services
AZ: availability zone
EMA: European Medicines Agency
FDA: US Food and Drug Administration
ICH-GCP: International Council for Harmonisation of Technical Requirements for Pharmaceuticals for Human Use–Good Clinical Practice
PMDA: Pharmaceuticals and Medical Devices Agency
SDV: source data verification
SQS: Simple Queue Service
